# A phase I study investigation of metabolism, and disposition of [^14^C]-anlotinib after an oral administration in patients with advanced refractory solid tumors

**DOI:** 10.1007/s00280-020-04062-8

**Published:** 2020-04-07

**Authors:** Yiqian Liu, Lianke Liu, Lingxiang Liu, Tongshan Wang, Lian Guo, Yixiang Wang, Zhengzhen Gao, Yongqian Shu

**Affiliations:** 1 Department of Oncology, First Affiliated Hospital of Nanjing Medical University, 300 Guangzhou Road, Nanjing, Jiangsu 210029 People’s Republic of China; 2 Department of DMPK Service, Lab Testing Division, WuXi AppTec Co. Ltd, Nanjing, 210038 China

**Keywords:** Anlotinib hydrochloride, Pharmacokinetics, Metabolism, Phase I study

## Abstract

**Purpose:**

Anlotinib is a novel oral multi-targeted receptor tyrosine kinase inhibitor, which selectively inhibits VEGFR2/3, FGFR1-4, PDGFR α/β, c-kit, and Ret. It shows antitumor effect in patients with advanced refractory solid tumors. The detailed absorption, metabolism, and excretion pathways of anlotinib have not yet been fully investigated.

**Methods:**

Six male patients were enrolled and divided into two groups. Group A (containing two patients) received 14.15 mg/80 µCi/subject [^14^C]-anlotinib hydrochloride. Group B (containing four patients) received 14.15 mg/120 µCi/subject [^14^C]-anlotinib hydrochloride. The blood, urine, and feces of all the six patients after orally administration of [^14^C]-anlotinib were collected. The absorption, metabolism, and excretion of [^14^C]-anlotinib were investigated, and the efficacy and safety of anlotinib were evaluated.

**Results:**

In plasma, the average time to peak concentration (*T*_max_) of total radioactivity was 4.42 h and the average peak concentration (*C*_max_) of total radioactivity was 18.80 ng Eq./g. The average values of AUC_0-last_, AUC_0-∞_, and MRT_0-t_ were 4071 h.ng Eq./g, 13,555 h.ng Eq./g, and 125 h, respectively. The average recovery of total radioactivity (TRA) in urine and feces was 62.03%, accounting for 48.52% and 13.51% in feces and urine of the total dosage, respectively. The parent drug, a carboxylic metabolite (M30), and mono-oxidation products (M46/M66) were major drug-related components in human plasma. Oxidative metabolism played the major role in drug clearance in human. The major metabolic pathways include oxidative deamination to M2, mono-oxidation to M1, and the formation of M30. Adverse events occurred in five patients and severe adverse events (SAE) occurred in one. Tumor response were evaluated as stable disease (SD) in three, partial response (PR) in one, and progressive disease (PD) in one of the patients, respectively.

**Conclusions:**

Anlotinib had a good pharmacokinetic profile with rapid absorption, long half-life, and extensive hepatic metabolism. The adverse events and efficacy were as expected.

**Electronic supplementary material:**

The online version of this article (10.1007/s00280-020-04062-8) contains supplementary material, which is available to authorized users.

## Introduction

Receptor tyrosine kinases (RTKs) are transmembrane glycoproteins that communicate with cellular growth factors and extracellular ligands [1]. RTKs have been implicated in a variety of pathological conditions, including cancer, metabolic and autoimmune disorders, infectious diseases, and neurodegenerative disorders [2]. RTK inhibitors have been successfully utilized in the treatment of several cancer types, most of which are multi-targeting such as imatinib, anlotinib, sorafenib, sunitinib, and pazopanib [3]. Anlotinib (1-[[4-(4-fluoro-2-methyl-1H-indol-5-yloxy)-6-methoxyquinolin-7-yl]oxy]methyl) cyclopropanamine dihydrochloride) is one of the newest developed oral small-molecule RTK inhibitors which has multiple targets including VEGFR1, VEGFR2/KDR, VEGFR3, c-Kit, PDGFR-α, and the fibroblast growth factor receptors (FGFR1, FGFR2, and FGFR3) [4]. The *vitro* studies using recombinant enzymes indicated that the capability of anlotinib to inhibit VEGFR2/KDR and VEGFR3 is approximately 20 and 500 times stronger compared with sunitinib and sorafenib [5]. The dysregulation of fibroblast growth factor (FGF)/FGF receptor axis results in aggressive cancer phenotypes by promoting cancer progression and enhancing the angiogenic potential of tumor microenvironment [6]. FGF/FGFR signaling alterations have also been found to be connected with chemotherapy resistance and poor prognosis. Preclinical studies have shown that anlotinib inhibits cell migration and formation of capillary-like tubes induced by VEGF/PDGF-BB/FGF-2 in endothelial cells [7].

Anlotinib suppresses tumor cell proliferation via inhibition of platelet-derived growth factor receptors α/β (PDGFR α/β), c-Kit, ret, as well as Aurora-B, c-FMS, and discoidin domain receptor 1 (DDR1), which are a group of newly identified kinase targets involved in tumor progression [8]. In addition, anlotinib showed antitumor activity against tumor cells carrying mutations in PDGFR α, c-Kit, Met, and epidermal growth factor receptor (EGFR) [9].

Anlotinib has been found to have a significant role in xenograft models in the renal, ovarian, colon, liver, glioma, and non-small cell lung cancer cells during dosing period [10]. The results of pharmacokinetic and disposition investigations in rats and dogs showed that anlotinib has good membrane permeability and is absorbed quickly. Metabolism studies in vitro and in rats demonstrated that anlotinib was primarily cleared via cytochrome P450-mediated hydroxylation and dealkylation. The oxidative metabolites were excreted directly to urine or bile or further converted to glucuronides or sulfates. Besides, anlotinib exhibited inhibitory activities against human cytochrome P450 3A4 and 2C9 in vitro with half maximum inhibitory concentrations of 0.11 μM and 0.25 μM, respectively [11].

Sun et al. performed a phase I study to determine DLT, MTD, basic pharmacokinetics, RP2D, and preliminary antitumor effects of anlotinib in patients with advanced refractory solid tumor. They found that anlotinib displayed manageable toxicity, long circulation, and broad-spectrum antitumor potential. The recommended treatment regimen for the subsequent clinical studies was as follows: anlotinib monotherapy; 12 mg per day on a 2/1 schedule [12].

The main objective of this phase I clinical study was to investigate the absorption, metabolism, and excretion of [^14^C]-anlotinib hydrochloride in patients after an oral administration. Plasma, urine, and feces were collected followed by total radioactivity analysis, radioactivity profiling, and metabolite structural characterization. Consequently, exposures to drug-related components, mass balance, and metabolic clearance pathways in human were determined.

## Materials and methods

### Study design

The phase I study of anlotinib was carried out in a single center and six male patients with advanced refractory solid tumor were enrolled. All the six male patients enrolled were divided into Group A (containing two patients) and Group B (containing four patients). The study was conducted in accordance with International Conference on Harmonization guidelines for Good Clinical Practice.

### Patients

#### Inclusion criteria

Patients who provided written consents aged between 18 and 70 years with histologically or cytologically confirmed advanced refractory solid tumors (including renal clear cell carcinoma, non-small cell lung cancer, soft-tissue sarcoma, and colorectal carcinoma), progressed from or relapsed after the first- or second-line treatment, were eligible. Other inclusion criteria included: (1) Eastern Cooperative Oncology Group (ECOG) Performance Score 0–1; (2) an estimated life expectancy > 3 months; (3) adequate bone marrow function (hemoglobin > 90 g/L, absolute neutrophil count(ANC) > 1.5 × 10^9^/L, platelet > 80 × 10^9^/L); (4) adequate hepatic function [total bilirubin < 1.5 times the upper limit of normal (ULN), alanine aminotransferase (ALT) and aspartate aminotransferase (AST) < 2.5 × ULN or < 5 × ULN for patients with liver metastases]; (5) adequate renal function [serum creatinine < 1.5 × ULN or creatinine clearance rate (CCR) > 30 mL/min]; (6) adequate cardiac function [left ventricular ejection fraction (LVEF) > 50%].

#### Exclusion criteria

The exclusion criteria included: (1) uncontrolled hypertension (systolic blood pressure > 150 mmHg, diastolic blood pressure > 100 mmHg with one antihypertensive drug); (2) urinary protein (+ +) and 24-h urinary protein quantification (> 1.0 g) was confirmed; (3) exposure to any drug that inhibits or induces drug metabolic enzymes within 30 days before study entry; (4) major surgical treatment, incision biopsy, or significant traumatic injury within 28 days; (5) arteriovenous thrombosis events within 6 months, such as cerebrovascular accident (including temporary ischemic attack), and deep vein thrombosis and pulmonary embolism; (6) large doses of radiation within 1 year, or radioactivity pharmacokinetic studies of human carbon 14 within 1 year.

The basic characteristics of patients are shown in Table 1.

**Table 1:** Baseline characteristics

Characteristics	Patients, *N* = 6
Sex, *n*	
Male	6
Female	0
Weight, median (range), Kg	67.6 (54.0–90.4)
Disease, *n*	
Anal ductal adenocarcinoma	2
Colon adenocarcinoma	1
Left parotid carcinoma	1
Abdominal liposarcoma	1
Lung adenocarcinoma	1
Lines of treatment, median (range)	3.5 (1–4)
AST, median (range), U/L	23.2 (14.1–129.0)
ALT, median (range), U/L	21.6 (16.8–110.4)
TBIL, median (range), umol/L	14.5 (7.6–17.6)
TC, median (range), mmol/L	5.2 (4.4–10.1)
TG, median (range), mmol/L	1.7 (0.5–2.5)
Cr, median (range), umol/L	76.6 (69.9–105.3)

### Study medication

[^14^C]-anlotinib hydrochloride suspension was prepared by XBL-China, Inc. (Department of DMPK Service, Lab Testing Division, WuXi AppTec Co. Ltd., Nanjing) The suspension containing anlotinib hydrochloride and [^14^C]-anlotinib hydrochloride which was prepared for orally administration of the patients contained 234 mg of excipients (including 89 mg mannitol, 138 mg microcrystalline cellulose, 6 mg hydroxypropyl cellulose, and 1 mg magnesium stearate). Patients in Group A administered a single oral dose containing 14.15 mg/80 µCi [^14^C]-anlotinib hydrochloride (totally equal to 12 mg of anlotinib). Patients in Group B administered a single oral dose containing 14.15 mg/120 µCi [^14^C]-anlotinib hydrochloride (totally equal to 12 mg of anlotinib).

### Sample collection

The blood samples were collected before orally administration and 0.5, 2, 4, 8, 10, 12, 24, 48, 72, 96, 120, 144, 168, 192, 216, 240, 288, 312, 384, 408, 456, 480, 552, 840, 888, and 1416 h after orally administration. An aliquot of the blood samples was mixed gently and chilled immediately under  − 20 °C; the remainder was centrifuged at 1660 g for 10 min at 4 °C to separate the plasma samples.

The urine samples were collected before orally administration and 0–2648 h after orally administration. The fecal samples were collected before orally administration and 0–1976 h after orally administration. In the process of collecting urine and feces samples of the six patients, because of the excretion particularity of the drug, the samples were not collected continuously every day, but once a week or every 10 days. In this case, the calculation of excretion rate during non-collected days was the multiplication of the first-day excretion rate after non-collected days by the number of non-collected days.

### Radioactivity dosimetry

A dosimetry analysis was conducted to estimate the human absorbed radiation dose of [^14^C] anlotinib hydrochloride-related radioactivity for oral administration of [^14^C] anlotinib hydrochloride. Total radiocarbon concentrations in organ/tissue distribution and excretion data obtained after oral administration of [^14^C] anlotinib hydrochloride to male rats were utilized for this analysis.

The animal data were subjected to relative organ mass-scaling and physiological time-scaling, and then fit with non-compartmental analysis using WinNonlin6.3 (Pharsight). The human-absorbed dose estimates were calculated following the MIRD (Medical Internal Radiation Dose) schema using the MIRDOSE3.1 software package (Oak Ridge Associated Universities, 1994) and manually in MicroSoft Excel for organs/tissues of interest that were not covered by MIRDOSE3.1.

For a human mass balance study involving radioactivity, the Effective Dose (ED) is typically kept below 1 mSv (Risk Category IIa or below) if possible. Based on the analysis presented herein, a single oral administration of 81 μCi or lower of [^14^C] anlotinib hydrochloride would predict an effective radiation absorbed dose at or below 1 mSv (Risk Category IIa, ICRP 62, one-fifth of the absorbed dose equivalent per patient of 5 mSv per year cited in the Swiss Radiological Protection Ordinance). In conclusion, a total dose of up to 81 μCi in humans is expected to result in a radiation exposure that is within the 100 mrem (1 mSv) acceptable absorbed dose limit in humans. 80 µCi/ subject was used for Group A, due to long half-life of anlotinib in humans, radioactivity in collected samples post-dose was near to background, and 120 µCi/ subject was used for Group B to obtain more precise data.

### Radioactivity determination

Levels of radioactivity in plasma, urine, and extracts of fecal samples were determined by mixing individual samples with 5 mL scintillation cocktail and determined by counting directly in a liquid scintillation counter (LSC). Blood, fecal homogenates, and post extraction solids were combusted with OX-501 Biological Sample Oxidizer, the ^14^CO_2_ formed were trapped directly in 15 mL scintillation cocktail, followed by counting with LSC. Samples were assayed with a liquid scintillation counter for 5 min.

### Sample preparation for radio-profiling and metabolite identification

#### Plasma

An equal volume of 2, 4, 8, 12, 24, and 96 h plasma sample was pooled by time-point across subject 03 ~ 06, to give six pooled plasma samples for metabolite profiling. An appropriate volume (3x, v/v) of acetonitrile was added to a portion of the pooled plasma sample, followed by vortexing ca. 2 min. After storage in a refrigerator for ca. 30 min, the mixture was centrifuged at ca. 10000 g at 4 °C for 10 min. The supernatant was then separated from the PES, which was suspended with water, followed by addition of methanol (2x, v/v) twice. The three extracts were combined and evaporated to dryness under a nitrogen stream by an N-Evap evaporator at room temperature. The residues were reconstituted with an appropriate volume of methanol:water (1:1, v/v) and centrifuged at ca. 10000 g at 4 ℃ for 10 min. The supernatant was subjected to HPLC radio-profiling.

#### Urine

Four pooled human urine samples (subject 03 ~ 06) were pooled from 0 to 432 h based on equal volume ratios from the individual patients across time intervals. In addition, three pooled urine samples (0–48, 48–120 and 360–432 h) (subjects 03–06) were prepared by pooling human urine samples across subjects and time points. Portion of the pooled urine sample was evaporated to a small volume under a nitrogen stream and extracted with methanol (3x, v/v) followed by centrifuged at ca. 10000 g at 4 ℃ for 10 min. The supernatant was reconstituted with an appropriate volume of methanol:water (1:1, v/v) and centrifuged at ca. 10000 g at 4 ℃ for 10 min. The supernatant was subjected to HPLC radio-profiling.

#### Feces

Four human fecal samples (0–432 h) were prepared by pooling feces from individual subjects (Subjects 03–06). In addition, three pooled human fecal samples (0–48, 48–120 and 360–432 h) were prepared by pooling feces samples across subjects and time points. The pooled fecal homogenate was extracted with methanol (3x, v/v) and the mixture was vortexed, and centrifuged at ca. 10000 g at 4 ℃ for 10 min. The supernatant was then separated from the PES, which was suspended with water, followed by addition of methanol twice. The three extracts were combined and evaporated to dryness under a nitrogen stream by an N-Evap evaporator at room temperature. The residues were reconstituted with an appropriate volume of methanol:water (1:1, v/v) and centrifuged at ca. 10000 g at 4 ℃ for 10 min prior to analysis.

### Bioanalytical assay

The calibration curve of anlotinib used for determination of anlotinib in LC–MS/MS contained eight calibrators. The linear range of the calibration curve was 0.868 ~ 111 nf/mL, and the concentrations of the eight calibrators were 0.868, 1.74, 3.47, 6.94, 13.88, 27.75, 55.5, and 111 ng/mL, respectively. The weighting factor was 1/*x*^2^.

### Parameters calculation

The peak concentration (*C*_max_) and the time to *C*_max_ (*T*_max_) were obtained through the plasma concentration directly. The area under concentration–time curve up to the last measured time point (AUC_0−*t*_) was calculated by the trapezoidal rule. The *k* was estimated by linear regression analysis of the terminal portion of the log concentration–time data, and the elimination half-life (t_1/2_) was calculated through the coefficient 0.693/k. The renal clearance (CLR) was calculated by dividing the cumulative amount excreted into urine (Cum. A_e−U_) by plasma AUC_0−*t*_.

### Metabolite profiling and characterization

HPLC coupled with radioactivity monitor (LC-RAM) was used to isolate and quantitate [^14^C] anlotinib and its metabolites. The plasma and fecal extracts as well as urine supernatants were subjected to HPLC separation, fractionation, and eluent collection. The radioactivity in each fraction was monitored by solid scintillation counting using a Packard TopCount® NXT™ Microplate Counter. The radio-profiles were then reconstructed using ARC® Convert software. The peak area of each radioactivity peak/region was integrated using ARC® Evaluation software, and the percent distribution of each peak/region was calculated (%TRA for plasma or %Dose for urine and feces).

Metabolites were characterized and/or identified by LC–MS/MS in conjunction with on-line radioactivity detection. Structures of metabolites were proposed by interpretation of the mass spectral fragmentation pattern, as well as chromatographic behaviors and metabolic pathways.

## Results

### Patients

Six patients with confirmed advanced refractory solid tumors were enrolled in May 2016. They had a mean weight of 70.4 kg (range from 54 to 90.4 kg) and a mean height of 170.55 cm (range from 164.8 to 175.6 cm). All patients had a history of anti-cancer drug therapy and anti-cancer surgery. At the time of enrollment, four patients (67%) had a WHO performance status score of 0 and two (33%) had 1.

### Pharmacokinetics

**Fig. 1:**
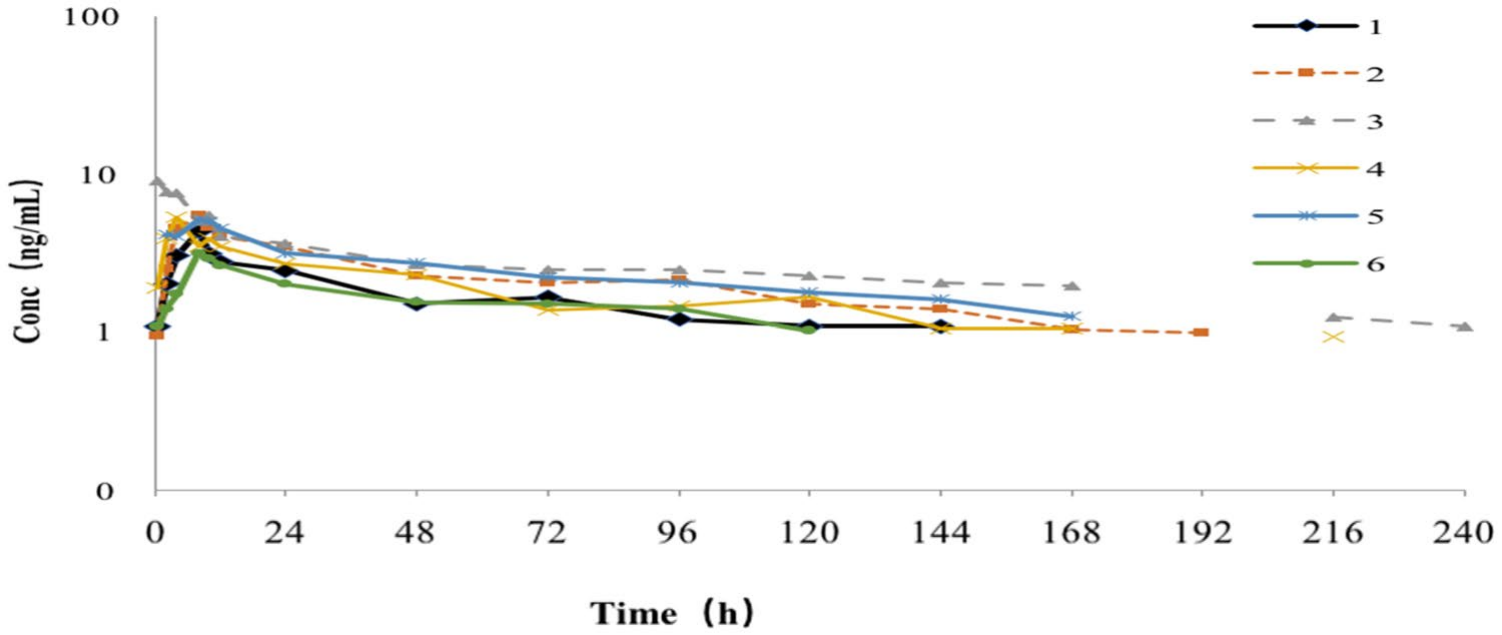
Plasma concentration–time curve of anlotinib in plasma samples from six subjects

Individual differences are significant after a single oral administration of 14.15 mg/80 μCi/human or 14.15 mg/120 μCi/human [^14^C]-anlotinib hydrochloride in male patients. The *T*_max_ of total radioactivity (TRA) in plasma is 4.42 h in average and the *C*_max_ of TRA is 18.80 ng Eq./g in average. The average values of AUC_last_ and AUC_0-∞_ are 4071 h∙ng Eq./g, 13,555 h∙ng Eq./g respectively, and the average value of MRT_0-t_ is 145 h. The plasma concentration–time curve of anlotinib in plasma samples from six patients is shown in Fig. 1.

### Mass balance

**Fig. 2:**
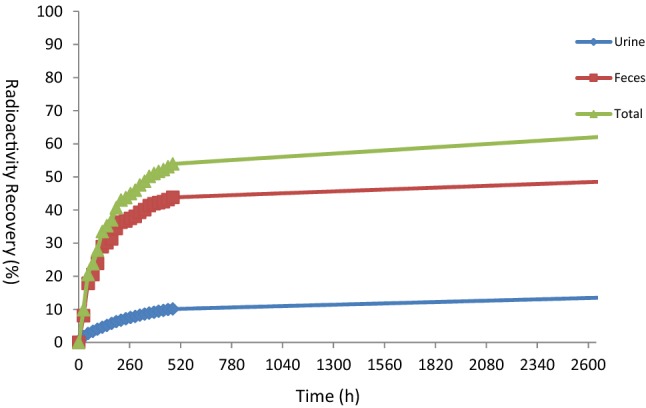
Mean cumulative excretion of radioactivity within 0–2642 h following a single oral dose of 14.15 mg/80 or 120 µCi/subject of [^14^C] anlotinib hydrochloride to male patients

For all of six patients, urine and fecal samples were collected during the 2648 h after administration of [^14^C]-anlotinib. Recovery of total radioactivity of the dose was 62.03%, of which 13.51% was from urine and 48.52% was from feces; feces was the major excretion route. The details are listed in Table 2. Mean cumulative excretion of radioactivity within 0–2648 h from urine and feces following a single oral dose of 14.15 mg/80 or 120 µCi /subject of [^14^C] anlotinib hydrochloride are shown in Fig. 2.

**Table 2:** [^14^C] Total recovery after 0 ~ 2648 h of anlotinib hydrochloride

Subject	Urine (% dose)	Feces (% dose)	Total recovery rate (% dose)
01	15.51	50.73	66.24
02	18.45	42.51	60.96
03	11.99	38.93	50.92
04	12.29	56.80	69.09
05	12.42	59.44	71.86
06	10.39	42.69	53.08
Average value	13.51	48.52	62.03
Standard deviation	2.94	8.42	8.59

### Metabolite detection and structural characterization

**Fig. 3:**
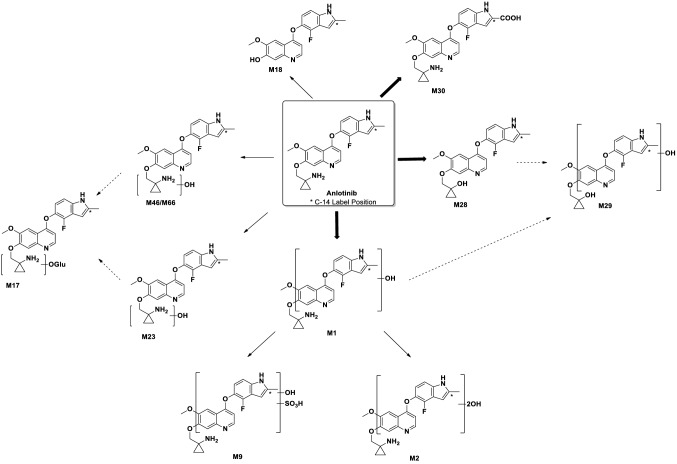
Proposed major metabolic pathways of anlotinib in human

In the current study, ten metabolites of anlotinib were found and structurally characterized in plasma and excreta in patients by LC-RAM/HRMS after administration of a single oral 14.15 mg/120 μCi/human of [^14^C]-anlotinib, which were designed as M1, M2, M9, M17, M18, M23, M28, M29, M30, and M46/M66 (Fig. 3). M1, M23, and M46/M66 were mono-oxidation products of anlotinib, M2 was di-oxidation product of anlotinib, M9 was a sulfate conjugate of a mono-oxidation product, M17 was a glucuronide conjugate of a mono-oxidation product, M18 was O-dealkylation product of anlotinib, M28 was an oxidative deamination product of anlotinib, M29 was a mono-oxidation product of M28, and M30 was oxidized to carboxylic acid product of anlotinib. Mass spectral data of these metabolites are summarized in Table 3.

**Table 3:** Spectral data of anlotinib metabolites

Metabolite	Identity	[M + H] + Da	MS^n^ fragments
Anlotinib	Parent drug	408.1718	339, 319, 304, 276, 248
M1	Mono-oxidative product of anlotinib	424.1668	355, 337, 320
M2	Di-oxidative product of anlotinib	440.1618	371, 327, 353, 264
M9	Sulfate conjugate of mono-oxidative product of anlotinib	504.1238	486, 435, 424, 355
M17	Glucuronide conjugate of mono-oxidative product of anlotinib	600.1996	424, 334, 406
M18	O-dealkyl product of anlotinib	339.1138	304, 276, 248
M23	Mono-oxidative product of anlotinib	424.1667	378, 339
M28	Oxidative deamination product of anlotinib	409.1558	391, 339, 337, 317, 289
M29	Mono-oxidative product of M28	425.1508	351, 339, 407
M30	Oxidized to carboxylic acid product of anlotinib	438.1459	369, 351
M46/M66	Mono-oxidative product of anlotinib	424.1666	339, 406

### Metabolite profiles of anlotinib in plasma, urine, and feces

- (1) Plasma
  The parent drug, M30, and M46/M66 were major drug-related component in human plasma (Supplementary Fig.1A). In addition, M1, M2, M9, M17, M18, M28, and M29 were detected in plasma. The identities of M1, M2, M9, M17, M18, M30, and M46/M66 were determined using LC–MS/MS analysis and the comparison with those in urine and feces. However, due to matrix interference and low concentration, M28 and M29 in plasma were not detected by LC–MS/MS. Their identities were determined by comparing their retention time with those in urine and feces. In addition, metabolites with high polarity (e.g., peaks 2 and 9) were detected in plasma samples, but their structures remained to be determined.
- (2) Urine
  The parent drug was one of the major drug-related components in urine (Supplementary Fig. 1B), accounting for 0.73% of the dose. M2, M9, M28, and M30 were the major metabolites in urine, accounting for 0.32% ~ 0.88% of the dose. M1, M17, M18, M23, M29, and M46/M66 were minor metabolites in urine, accounting for 0.07% ~ 0.25% of the dosage. In addition, highly polar metabolites (e.g., peak 2, 9, 10, and 11) were detected in urine samples, but their structures remained to be determined. Other metabolites were less than 0.40%.
- (3) Feces
  The parent drug was one of the major drug-related components, accounting for 5.64% of the dose (Supplementary Fig. 1C). M9, M28, and M30 were major metabolites in feces, accounting for 2.97% ~ 5.91% of the dosage; M1, M2, M17, M18, M23, M29, and M46/M66 were minor metabolites in feces, accounting for 0.20% ~ 2.57% of the dosage. Other metabolites accounted for less than 1.20% of the dose.
- (4) Metabolic pathways
  The major metabolic routes of anlotinib in human include: (1) oxidative deamination to M28, (2) mono-oxidation to M1, followed by further oxidation to M2 and sulfate conjugation to M9, and (3) oxidation to carboxylic acid product (M30). Minor metabolic routes include: (1) mono-oxidation to M23, M46/M66, followed by glucuronide conjugation to M17 and (2) O-dealkylation to M18. The metabolic pathways of anlotinib in human are proposed in Fig. 3.

## Safety

After a single dosage, adverse events occurred in five patients, while SAE occurred in one during the follow-up period. Treatment-related grade 3 adverse events occurred in the course of drug use. The frequency of adverse events was sorted as following: increased lipase, increased bilirubin, increased alanine aminotransferase, hypophosphatemia, increased gamma-glutamyltransferase, increased aspartate aminotransferase, and liver function damage. Grade 1/2 adverse events included increased lipase, hypoalbuminemia, increased bilirubin, constipation, proteinuria, etc. The details are listed in Table 4.

**Table 4:** Adverse events occurred throughout the study after a single dose of 14C anlotinib suspension

Adverse events	Number of cases of grade 1/2 (%)		Number of cases of grade 3 (%)	
*N* = 6* n* (%)	D1 ~ D18	Research process	D1 ~ D18	Research process
At least once	5 (83.33%)	5 (83.33)	3 (50%)	3 (50%)
Increased lipase	2 (33.33%)	2 (33.33%)	2 (33.33%)	2 (33.33%)
Increased blood bilirubin	1 (16.67%)	1 (16.67%)	1 (16.67%)	1 (16.67%)
Increased alanine aminotransferase	1 (16.67%)	1 (16.67%)	1 (16.67%)	1 (16.67%)
Hypophosphatemia	1 (16.67%)	1 (16.67%)	1 (16.67%)	1 (16.67%)
Increased γ-glutamyl transpeptidase	1 (16.67%)	1 (16.67%)	1 (16.67%)	1 (16.67%)
Aspartate aminotransferase increased	0	0	1 (16.67%)	1 (16.67%)
Liver function damage	0	0	1 (16.67%)	1 (16.67%)
Hypoalbuminemia	3 (50%)	3 (50%)	0	0
Constipation	2 (33.33%)	2 (33.33%)	0	0
Proteinuria	1 (16.67%)	1 (16.67%)	0	0
Hypocalcemia	1 (16.67%)	1 (16.67%)	0	0
Sinus tachycardia	1 (16.67%)	1 (16.67%)	0	0
Sinus bradycardia	1 (16.67%)	1 (16.67%)	0	0
Diarrhea	1 (16.67%)	2 (33.33%)	0	0
Hypertriglyceridemia	1 (16.67%)	1 (16.67%)	0	0
Hypothyroidism	1 (16.67%)	1 (16.67%)	0	0
Alkaline phosphatase increased	1 (16.67%)	1 (16.67%)	0	0
Cough	1 (16.67%)	1 (16.67%)	0	0
Rapid atrial fibrillation	1 (16.67%)	1 (16.67%)	0	0
Anemia	1 (16.67%)	1 (16.67%)	0	0
Increased lactate dehydrogenase	1 (16.67%)	1 (16.67%)	0	0
Stomachache	1 (16.67%)	1 (16.67%)	0	0
Insomnia	2 (33.33%)	2 (33.33%)	0	0
Hemoptysis	0	1 (16.67%)	0	0

## Discussion

In this study, we found that the average recovery of TRA in urine and feces was 62.03%. Prototype drugs and metabolites were mainly excreted through feces and urine, accounting for 48.52% and 13.51% of the total dosage. The low recovery of TRA might result from four reasons: first, half-life period for the elimination of anlotinib in human body is long. After absorbed into human circulatory system, the slow excretion of anlotinib resulted in a relatively low concentration of radioactivity in samples (radioactive concentration after 404–432 h is 279 DPM/ml in urine and 4876 DPM/ml in feces), which might affect the low radioactivity; second, a single dosage of 14.15 mg/80uCi/human or 14.15 mg/120uCi/human [^14^C]-anlotinib hydrochloride suspension was administered followed by anlotinib (12 mg for 2 consecutive weeks followed by 1 week discontinuation) after 432 h (504 h for Subject 04). As a result, [^14^C]-anlotinib hydrochloride and its metabolites were diluted and redistributed after the large quantity of unlabeled drugs entered circulation, reducing the excretion rate of labeled drugs and their metabolites [13]; third, in the process of collecting urine and feces samples of the six patients, because of the excretion particularity of the drug, the samples were not collected continuously every day, but once a week or every 10 days. In this case, the calculation of excretion rate during non-collected days was the multiplication of the first-day excretion rate after non-collected days by the number of non-collected days. This calculation method might lead into errors; fourth, other metabolic pathways, such as breath and sweat, might lower the recovery.

We found that *C*_max_ and AUC were twice as much as the results in published phase I study, which might result from two reasons. One is the difference of drug administration. Capsule was used in the previous study, while suspension was adapted in our study in considering of the configuration and use of radiopharmaceuticals. The other is the small sample size of only six patients in this study versus 11 in the previous study. The difference was acceptable considering the fixed dosage of drug with individual differences (such as height, weight, or the activity of P450 metabolic enzymes) [14].

The plasma concentration of anlotinib hydrochloride was analyzed by LC–MS/MS. All of the data were in accordance with the standard operating procedures of Nanjing Mesino Pharmaceutical Technology Co., Ltd. The results showed that TRA concentration in plasma and blood was low after [^14^C]-anlotinib hydrochloride administration, and the individual difference was significant.

The liver was the main excretory organ and the kidney was the secondary excretory organ after a single dosage of [^14^C]-anlotinib hydrochloride. The main clearance method of anlotinib hydrochloride was considered to be metabolized by phase I enzyme and then excreted from liver to feces. Besides, the direct excretion of anlotinib from the kidney is also another important way of clearance. According to metabolite identification of [^14^C]-anlotinib hydrochloride, the main metabolic pathways of anlotinib hydrochloride include: (1) amino oxidation (amino to hydroxyl) to form M28; (2) mono-oxidation to form M1, and further to oxidative metabolite (M2) and sulfuric acid conjugate (M9); (3) carboxylation to form M30. Additionally, the secondary metabolic pathways of anlotinib hydrochloride in the patients including: (1) mono-oxidation to form M23 and/or M46/M66 and further convert to glucuronic acid-binding product (M17); (2) O-dealkylation to form M18 [15].

Patients were well tolerated to single dosage of 14.15 mg/80 µCi/human or 14.15 mg/120 µCi/human [^14^C]-anlotinib hydrochloride suspension during the observational period, and most of them presented with grade 1/2 adverse events. It is suggested that the safety of patients could be guaranteed with a single dosage of [^14^C]-anlotinib hydrochloride suspension (14.15 mg/80 µCi or 14.15 mg/120 µCi).

## Conclusions

In conclusion, anlotinib showed a pharmacokinetic profile of rapid absorption, long half-life, and extensive hepatic metabolism. The efficacy was promising with a favorable toxicity profile as expected, demonstrating that anlotinib could be further investigated in clinic.

## Electronic supplementary material

Below is the link to the electronic supplementary material.

Supplementary file1 (DOCX 297 kb)
