# Supplementary material for: A phase I study investigation of metabolism, and disposition of [14C]-anlotinib after an oral administration in patients with advanced refractory solid tumors
Source: Cancer Chemother Pharmacol. 2020 Apr 7;85(5):907–15. doi: 10.1007/s00280-020-04062-8 (PMC7188728; doi:10.1007/s00280-020-04062-8)
Supplement: Supplementary file 1 — Supplementary file1 (DOCX 297 kb) [file 280_2020_4062_MOESM1_ESM.docx]

Supplementary Figure 1. Representative LC-Radioprofiles of Plasma (A), Urine (B) and Feces (C) Samples

**
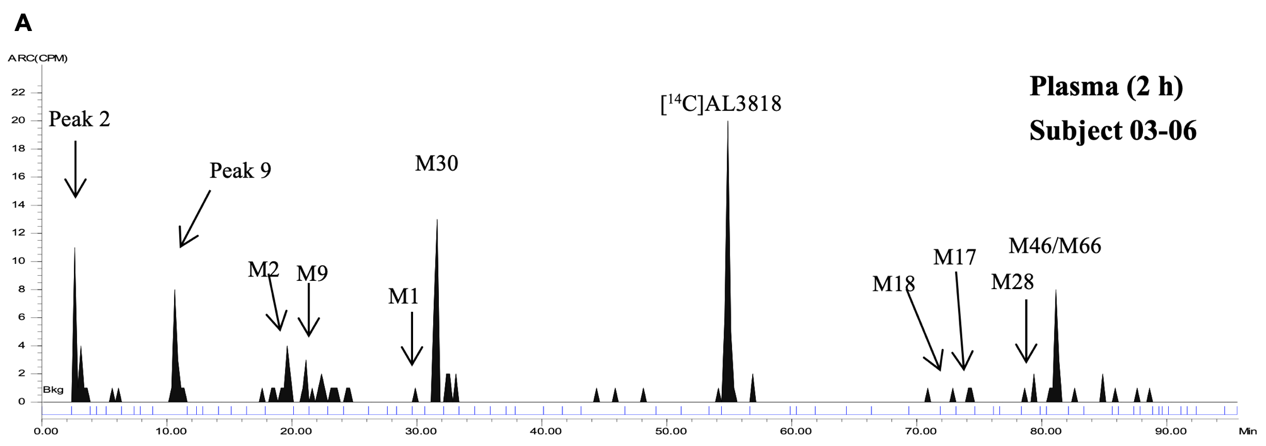
**


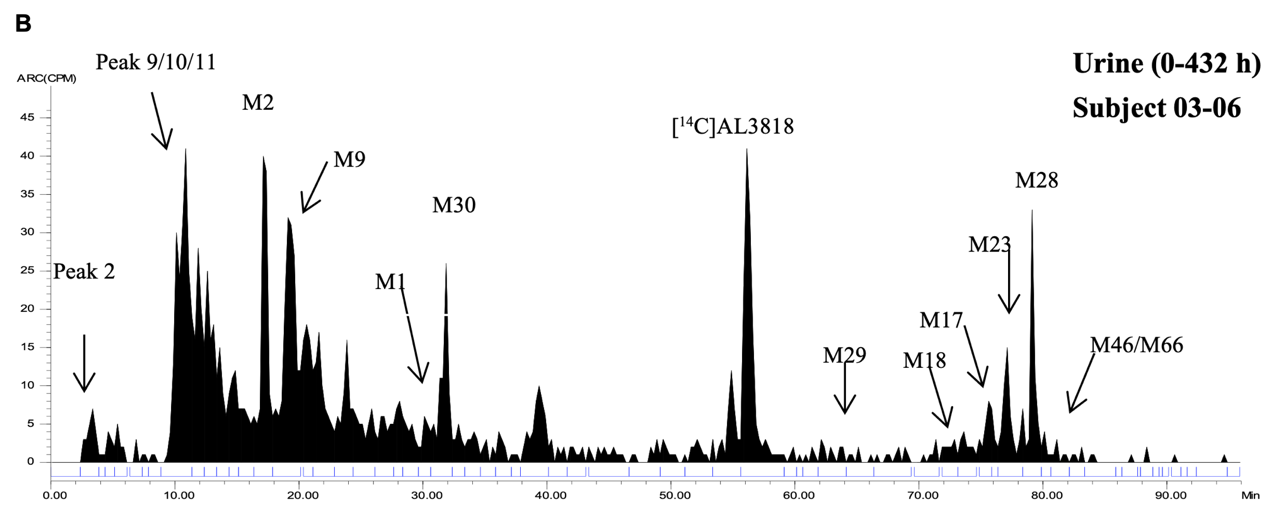


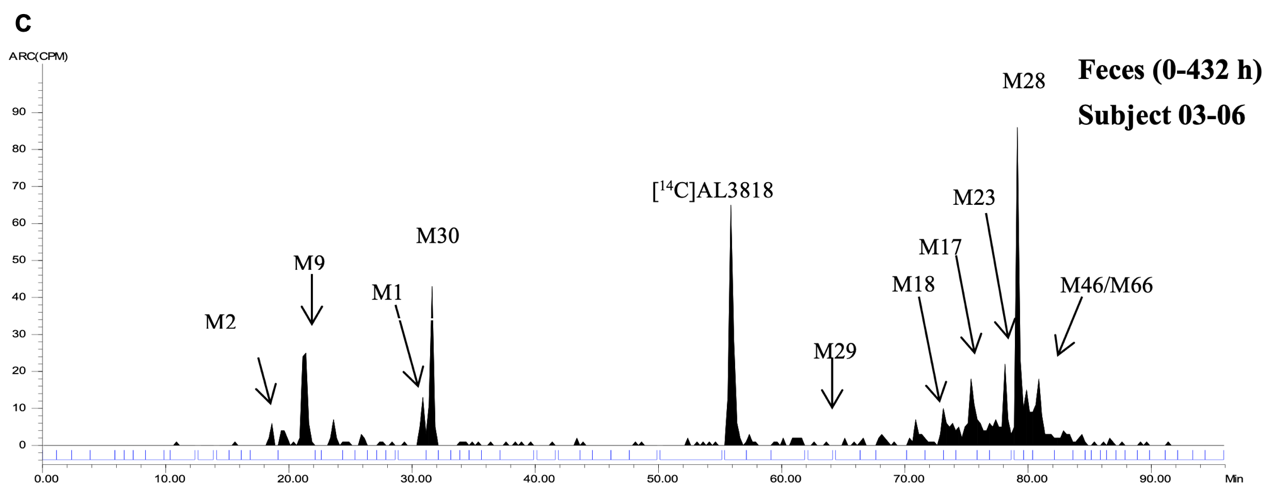


AL3818: Anlotinib
